# Comparison of machine learning methods for prediction of osteoradionecrosis incidence in patients with head and neck cancer

**DOI:** 10.1259/bjr.20200026

**Published:** 2021-03-18

**Authors:** Laia Humbert-Vidan, Vinod Patel, Ilkay Oksuz, Andrew Peter King, Teresa Guerrero Urbano

**Affiliations:** 1 Guy's and St Thomas' Hospital NHS Foundation Trust, London, UK; 2 School of Biomedical Engineering & Imaging Sciences, King’s College London, London, UK; 3 Computer Engineering Department, Istanbul Technical University, Istanbul, Turkey; 4 Clinical Academic Group, King’s College London, London, UK

## Abstract

**Objectives:**

Mandible osteoradionecrosis (ORN) is one of the most severe toxicities in patients with head and neck cancer (HNC) undergoing radiotherapy (RT). The existing literature focuses on the correlation of mandible ORN and clinical and dosimetric factors. This study proposes the use of machine learning (ML) methods as prediction models for mandible ORN incidence.

**Methods:**

A total of 96 patients (ORN incidence ratio of 1:1) treated between 2011 and 2015 were selected from the local HNC toxicity database. Demographic, clinical and dosimetric data (based on the mandible dose–volume histogram) were considered as model variables. Prediction accuracy (measured using a stratified fivefold nested cross-validation), sensitivity, specificity, precision and negative predictive value were used to evaluate the prediction performance of a multivariate logistic regression (LR) model, a support vector machine (SVM) model, a random forest (RF) model, an adaptive boosting (AdaBoost) model and an artificial neural network (ANN) model. The different models were compared based on their prediction accuracy and using the McNemar’s hypothesis test.

**Results:**

The ANN model (77% accuracy), closely followed by the SVM (76%), AdaBoost (75%) and LR (75%) models, showed the highest overall prediction accuracy. The RF model (71%) showed the lowest prediction accuracy. However, based on the McNemar’s test applied to all model pair combinations, no statistically significant difference between the models was found.

**Conclusion:**

Based on our results, we encourage the use of ML-based prediction models for ORN incidence as has already been done for other HNC toxicity end points.

**Advances in knowledge:**

This research opens a new path towards personalised RT for HNC using ML to predict mandible ORN incidence.

## Introduction

Radiotherapy (RT) is one of the main treatments for head and neck cancer (HNC), either alone or combined with chemotherapy or surgery. Ionising radiation damages tumour cells but it also affects the surrounding healthy tissue. Normal tissue toxicity can result in severe complications, and therefore limits the radiation doses that can be safely delivered to the patient. Concomitant chemoradiation improves local control and survival rates,^1^ but it can also result in toxicities such as mucositis, xerostomia, dysphagia or mandibular osteoradionecrosis (ORN).

Mandibular ORN is perhaps not the most frequent (5–15% prevalence rate),^2^ but certainly one of the most severe complications in patients with HNC undergoing radiation therapy. In particular, patients with oropharyngeal cancer seem to have a higher incidence of ORN.^3^ Ionising radiation damages the mucosa and vascularity of the mandible. Any trauma (*e.g.* surgery or dental extractions) caused to a previously irradiated mandible has a higher risk of resulting in necrosis of the bone due to poorer healing capabilities caused by decreased blood supply to that region.^4^ Factors of risk for the development of mandibular ORN include oral hygiene, drinking alcohol, smoking tobacco,^5^ dental extractions,^6,7^ mandibular surgery^8^ and high radiation doses to the mandible bone.^4,8,9^

Although much published work has focused on investigating the risk factors associated with mandible ORN, the prediction of mandible ORN incidence on a case-by-case basis is still subject to significant uncertainty.

The traditional approach to predicting clinical complications resulting from RT involves the use of Normal Tissue Complication Probability (NTCP) models, which have evolved over the years from the Lyman–Kutcher–Burman model^10^ to include clinical and demographic factors in addition to dosimetric parameters.^11^ Univariate and multivariate logistic regression (LR) models have been widely used to find correlations between the incidence of mandible ORN and clinical and dosimetric risk factors.^4,5,9^ More complex supervised machine learning (ML) methods, including support vector machines (SVM), random forest (RF), adaptive boosting (AdaBoost) and artificial neural networks (ANN), have been used to develop NTCP models for clinical decision-support^12^ in HNC RT.^13–16^ However, these do not include mandible ORN in the toxicity end points considered.

This paper compares the performance of different ML methods, including LR, SVM, RF, AdaBoost and ANN, in the prediction of mandibular ORN incidence.

## Methods and materials

### Patient selection

Study cases were selected from a database of patients with HNC treated with RT maintained by the local clinical oncology team between 2011 and 2015 (institutional approval 21 April 2016, project number 6333). A total of 48 HNC patients with ORN (28 oropharynx, 13 oral cavity, 3 larynx, 1 paranasal sinus and 3 unknown primaries) and 48 HNC control cases (28 oropharynx, 9 oral cavity, 7 larynx, 2 hypopharynx, 1 paranasal sinus and 1 unknown primary) were selected from the HNC database. Prescribed radiation doses ranged between 50 Gy in 20 fractions and 71.5 Gy in 30 fractions. Low-dose palliative cases, cases that were not treated with intensity-modulated radiation therapy (IMRT), and cases with incomplete data were excluded. Table 1 provides a summary of the demographic and clinical characteristics considered in this study.

**Table 1.** Summary of demographic and clinical characteristics of the two patient groups (ORN and non-ORN) in the study cohort

	ORN^a^	non-ORN
Gender (male)	34 (71%)	38 (79%)
Age (median)	64	59
Smoking (at RT start)	25 (52%)	19 (40%)
Smoking (previous)	14 (29%)	19 (40%)
Alcohol (at RT^b^ start)	33 (69%)	33 (69%)
Alcohol (previous)	5 (10%)	4 (8%)
Chemotherapy	33 (69%)	33 (69%)
Dental extractions (pre-RT)	30 (63%)	31 (65%)
Dental extractions (post-RT)	5 (10%)	2 (4%)
Surgery (pre-RT)	10 (21%)	18 (38%)
Surgery (post-RT)	1 (2%)	3 (6%)
Tumour site		
Oropharynx	28 (58%)	28 (58%)
Larynx	3 (6%)	7 (15%)
Oral cavity	13 (27%)	9 (19%)
Hypopharynx	0 (0%)	2 (4%)
Paranasal sinus	1 (2%)	1 (2%)
Unknown	3 (6%)	1 (2%)

a Osteoradionecrosis.

b Radiotherapy.

### Patient follow-up and definition of ORN

The local protocol for HNC patients includes clinical follow up for 5 years. Treatment outcomes are assessed with regards to tumour control and normal tissue complications. Patients who develop ORN at our centre are treated and closely monitored by a specialist oral surgical team in a dedicated clinic. The Radiation Therapy Oncology Group (RTOG) Late Radiation Morbidity Scoring Schema for bone tissue and the Notani^17^ ORN classification system were used in this study to score ORN toxicity grade at presentation, 3 months, 6 months, 12 months and yearly follow-up time points. The median time from the end of RT to diagnosis of ORN was 7 months (Tsai et al^9^ observed a median time of 8 months to develop ORN in their cohort). The minimum follow-up time in our control group was 13 months. For the purpose of this binary classification study, toxicity outcome was dichotomised and any grade of ORN was considered as an ORN case.

### Data preparation

Following local clinical practice, planned dose was recalculated using the dose-to-medium calculation method for those cases that were originally calculated using the dose-to-water approach. A copy of the original treatment plan was used to change the treatment planning system dose calculation settings from dose-to-water to dose-to-medium. Any CT image artefacts around the mandible area (*e.g.* metal artefacts or beam hardening) were corrected by overwriting the CT numbers (*i.e.* material density) prior to dose recalculation. The mandible bone was manually contoured and the mandible dose-volume histogram (DVH) data exported from the Monaco treatment planning system (TPS) for each patient. Dose-volume data were corrected for fractionation scheme differences to a standard fraction size of 2 Gy (*i.e.* EQD2) assuming an alpha-beta ratio of 3 for late effects. Maximum (Dmax) and mean (Dmean) mandible doses and relative dose–volume levels in the range V40 Gy to V70 Gy in 5 Gy increments were considered as the DVH-based dosimetric variables. Demographic and clinical variables considered are shown in Table 1.

### Variable selection

Univariate analysis using the χ^2^ significance statistical test was applied to the categorical variables considered for variable selection. The discriminatory power of the continuous variables (*i.e.* age and DVH-based dosimetric variables) was assessed with the Mann–Whitney *U* non-parametric test.^18^

### Nested cross-validation

Using the same data sets for hyperparameter optimisation and model selection can result in overfitting. In order to avoid this, stratified nested k-fold cross-validation (CV) was used in all models with *k* = 5 for both the inner and outer CV folds. In addition, for the ANN model, stratified data split was applied at each CV fold to the training data set to obtain the final training (80%) and internal validation (20%) data sets in the model selection process. Hyperparameter optimisation was done using the sklearn GridSearchCV function. The average accuracy across all inner CV folds was calculated for each hyperparameter combination and the results reported are for the outer CV folds using the hyperparameter combination that obtained the highest average accuracy.

### Model design and training

The predictive accuracy of four supervised ML methods (multivariate LR, SVM, RF and GB) was tested using the SciKit-Learn package in Python 2.7.15.^19^

The multivariate LR model was implemented using Python’s Scikit-Learn logistic regression classifier module (sklearn.linear_model.LogisticRegression) with a C parameter of 0.001 and the “l2” regularisation penalty. The SVM classifier^20^ was implemented using Python’s sklearn SVC class with a radial basis function (rbf) kernel, a penalty parameter (C) of 100 and a γ parameter of 0.001. The RF classifier^21^ was implemented using the sklearn.ensemble. Random Forest Classifier class with a maximum number of estimators of 10, a maximum tree depth of 50, a minimum number of samples at a leaf node of 1 and a minimum number of samples required to split an internal node of 0.5. The AdaBoost classifier^22^ was implemented using the sklearn.ensemble.AdaBoostClassifier class with a learning rate of 0.0001 and a maximum number of estimators of 10.

The ANN was implemented in Keras with Tensorflow as the backend and trained on a Nvidia Titan Xp GPU. It consisted of an input layer with the number of input nodes equal to the number of variables used, followed by a 200-node hidden dense (fully connected) layer with the rectified linear unit (ReLU) activation function and a 1-node output layer with the sigmoid activation function for binary classification (ORN or not ORN). A dropout layer was added at the end of the network pipeline to reduce overfitting.

The Binary Cross-Entropy loss function was used to train the ANN and the Adam optimiser was used to minimise the loss function. Based on the grid search results, the model was trained for 2000 epochs with a batch size of 30, a dropout rate of 0.0 and a learning rate of 0.001. The best model was chosen based on the highest accuracy achieved with the validation data set during training.

## Results

### Variable selection

The performance of the models was generally enhanced (Table 2) when using only the most statistically significant variables as per the variable selection process. Based on the univariate analysis with the χ^2^ significance statistical test using a *p*- value of 0.3, dental extractions post-RT (*p* = 0.26) and surgery pre-RT (*p* = 0.13) were included as clinical variables. The Mann–Whitney *U* non-parametric test was applied to all continuous variables with a level of significance of 0.05 and showed that age (*p* = 0.115) failed to reject the null hypothesis while Dmax (*p* = 0.000003) and Dmean (0.0002) were found to be the only dosimetric variables with class discriminating power. Table 2 shows that the prediction performance of all models improved when trained on the selected variables only.

**Table 2.** Comparison of prediction performance obtained with the five models considered: multivariate LR, SVM, RF, AdaBoost and ANNa

Model	Variables	Performance measure					
		Training accuracy	Testing accuracy	TPRb	TNRc	PPVd	NPVe
LR	All	0.76	0.73	0.98	0.47	0.66	0.97
	Selected	0.77	0.75	0.90	0.60	0.71	0.88
SVM	All	0.88	0.67	0.75	0.60	0.65	0.71
	Selected	0.84	0.76	0.96	0.56	0.68	0.94
RF	All	0.77	0.60	0.63	0.56	0.59	0.61
	Selected	0.78	0.71	0.77	0.66	0.70	0.76
AdaBoost	All	0.78	0.74	0.96	0.52	0.67	0.92
	Selected	0.78	0.75	0.93	0.56	0.68	0.91
ANN	All	0.71	0.66	0.75	0.57	0.66	0.68
	Selected	0.88	0.77	0.90	0.64	0.72	0.90

ANN, artificial neural network; AdaBoost, Adaptive Boosting; LR, logistic regression; RF, random forest; SVM, support vector machine.

Results including all variables and only the selected variables (Dmax, Dmean, extractions post-RT and surgery pre-RT) are shown.

a The figures shown correspond to the average over the five stratified cross-validation folds.

b True positive rate (sensitivity or recall).

c True negative rate (specificity).

d Positive predictive value (precision).

e Negative predictive value.

### Model performance comparison

For all models, at each of the 5 outer cross-validation folds, 20 cases were kept unseen by the model for testing its performance. The remaining 76 cases were used for model training in the LR, SVM, RF and AdaBoost models. For the ANN model, the training data set was further split during training into 60 cases for model training and 16 for model validation (80:20). Model performance was assessed using the following measures (Table 2): test accuracy (proportion of correctly predicted output class cases within the test set), true positive rate (TPR, also known as sensitivity or recall), true negative rate (TNR, also known as specificity), positive predictive value (PPV, also known as precision) and negative predictive value (NPV).

Based on these results (Table 2), no single model outperforms the rest in all measures considered. The ANN model (77%), closely followed by the SVM (76%), LR (75%) and AdaBoost (75%) models, has the highest overall prediction accuracy on an unseen test dataset. The McNemar’s statistical hypothesis test was used to determine whether the models compared agree or disagree in the same way. After a Bonferroni correction for multiple comparisons was applied, the significance threshold was set to 0.005. The test results showed that there is no statistically significant difference between the models (Table 3).

**Table 3.** Results from the McNemar’s statistical hypothesis test on all model pair combinations

χ^2^ (*p* value)	LR^*b*^	SVM^*a*^	RF^*c*^	AdaBoost^*d*^
SVM *vs*.	4.000 (0.754)			
RF *vs*.	5.000 (0.424)	7.000 (0.263)		
AdaBoost *vs*.	5.000 (1.000)	3.000 (1.000)	4.000 (0.267)	
ANN^*e*^ *vs*.	6.000 (0.607)	3.000 (1.000)	7.000 (0.189)	6.000 (0.791)

The first number is the McNemar’s test statistic (χ^2^) and the number in brackets is the corresponding *p* value (probability of observing this, or a larger, χ^2^ value). After a Bonferroni correction for multiple tests, a significance threshold of 0.005 was used. In all model pairs, the tests failed to reject the null hypothesis that the performance of the compared models is equal.

a Logistic Regression (LR)

b Support Vector Machine (SVM)

c Random Forest (RF)

d Adaptive Boosting (AdaBoost)

e Artificial Neural Network (ANN)

## Discussion

Most ORN-related published work has focused on finding correlations between ORN incidence and clinical and dosimetric variables based on population studies. While it is important to understand these associations, the ability to predict incidence on a case-by-case basis would be a more valuable clinical application. This study presents a clear performance comparison between ML prediction models for mandible ORN incidence. The prediction accuracies obtained show that ML-based methods can be used to assist clinical decision-making for HNC patients undergoing RT. We cannot recommend a specific model based on our prediction performance results, as these were not found to be statistically significantly different. The ANN model showed the highest overall prediction accuracy, but it could be argued that this is the model with poorest interpretability (even though there are tools that can enable some interpretable analysis on ML predictions).^23^ A simpler and more transparent model (*e.g.* LR) might be preferred for clinical use if the prediction performance is not significantly compromised.

Mandible ORN is a rare toxicity and the data sets available are naturally small. Low patient numbers make it difficult to attempt a multiclass prediction task where not only incidence but also ORN severity is predicted. The morbidity caused by ORN (at any grade) is such that the prediction of its incidence alone would already be an important clinical decision-support contribution. However, future work will aim at increasing the size of the study cohort.

Overfitting and poor generalisability are common problems when testing complex ML and deep learning models on independent data sets. We used a fivefold nested cross-validation scheme in our evaluation and none of the test data were used when training or tuning hyper-parameters for the models that were applied to them. This represents a fair internal validation of the ML models.

According to the TRIPOD statement,^24^ prediction studies can be classified into three categories: model development, model validation or a combination of both. The work presented falls into the first category, where we describe the first steps towards the development of a mandible ORN incidence prediction model by comparing different ML models. Future work will aim at the clinical implementation of a mandible ORN incidence prediction model. For this, external validation of the results on an entirely independent data set will be included.

The next steps of this study are focused on developing a deep neural network in order to include other variable types such as images and spatial dose information. ML-based approaches have previously exploited spatial dose information by using voxel-based (VB) methods to extract radiation dose metrics from the dose distribution maps as features to be used as input variables in a ML algorithm, usually together with clinical and demographic variables.^14,25^ The use of convolutional neural networks (CNNs), although widely employed in medical imaging applications, has yet to be fully explored for toxicity prediction. An advantage of using a CNN instead of a VB method is that the interpatient image registration may not be needed in the former, as the network is in principle capable of learning such variations from its training data. Moreover, a CNN is able to learn the image-based features that are most useful for the task being trained for. Men et al^26^ have recently showed this based on CT images and dose distribution volumes for the prediction of xerostomia in patients with HNC treated with RT.

The median Dmax was 69.0 Gy (range 57.6–74.6 Gy) and 58.5 Gy (range 23.3–74.8 Gy) for the ORN and control groups, respectively. Chen et al^4^ found that a total dose higher than 75 Gy resulted in a greater risk of ORN for patients with oral cancer. Even though our median Dmax is lower than the dose cut-off they suggest, we also see a correlation between high radiation doses to the mandible bone and incidence of ORN. However, the median Dmean within our ORN group was lower than that of the control group. Although initially surprising, this could highlight the need to reduce radiation dose “hot spots” within the mandible bone dose distribution. A study published by the MD Anderson Head and Neck Cancer Symptom Working Group^27^ found exactly the opposite and they observed a higher mandibular Dmean in the ORN group. Their study included oropharynx cancer patients only while our study includes a much larger variety of HNC sites resulting in a less homogeneous RT treatment range.

The International Commission on Radiation Units and Measurements (ICRU) Report 83^28^ recommends the transition from single spatial-point reporting (*e.g.* maximum and minimum absorbed doses, Dmax and Dmin, respectively) to dose–volume reporting (*e.g.* near-maximum and near-minimum absorbed doses, D2% and D98%, respectively). In this study, we used single spatial-point metrics because most existing literature report the mandible high dose tolerance as Dmax and comparison of results was easier. However, we do acknowledge that the transition towards dose–volume reporting metrics is necessary as these are more relevant to the IMRT era.

## Conclusion

This research aims to open a new path towards personalised RT for HNC using ML to predict mandible ORN incidence. We propose a new approach in the field of mandible ORN toxicity by using a prediction model for its incidence on a case-by-case basis rather than just determining its potential contributing factors. We have shown that this can be successfully done using ML methods. We therefore encourage the transition to ML-based prediction models for ORN as has already taken place for other HNC toxicity end points.
